# Cultural Validation of the Fear-of-Intimacy Scale for the Portuguese Population: Exploring Its Relationship with Sociosexual Orientation

**DOI:** 10.3390/ijerph22020274

**Published:** 2025-02-13

**Authors:** Ângela Leite, Ângela Azevedo

**Affiliations:** Centre for Philosophical and Humanistic Studies, Faculty of Philosophy and Social Sciences, Universidade Católica Portuguesa, 4710-362 Braga, Portugal; amazevedo@ucp.pt

**Keywords:** fear of intimacy, sex assigned at birth, romantic relationship, sociosexual orientation, casual sex

## Abstract

Objective: This study aims to adapt the Fear-of-Intimacy Scale for the Portuguese population and examine its association with sociosexual orientation, as measured by the Sociosexual Orientation Inventory, highlighting the relevance of this association as a public health issue. Methods: The Fear-of-Intimacy Scale was validated through confirmatory factor analyses. A multigroup analysis, employing confirmatory factor analysis, was conducted to evaluate the consistency of the Fear-of-Intimacy Scale across individuals in and out of romantic relationships. The reliability of the model was assessed using various indicators, including Cronbach’s alpha, McDonald’s omega, composite reliability, the average variance extracted (AVE), and the square root of the AVE. Results: The results indicated robust psychometric properties for the Fear-of-Intimacy Scale, with a well-fitting model identified. Configural, metric, and scalar invariance related to being in a romantic relationship or not were established; however, error variance invariance was not achieved. Although most dimensions of the two instruments displayed positive and significant relationships, the correlation values were generally modest. Conclusion: The findings underscore the importance of understanding the relationship between fear of intimacy and sociosexual orientation within the Portuguese context as a significant public health issue. Adapting the Fear-of-Intimacy Scale enables culturally sensitive research and supports clinical interventions aimed at improving relational and psychological well-being, thereby addressing broader public health challenges.

## 1. Introduction

Adapting the Fear-of-Intimacy Scale for the Portuguese population facilitates culturally sensitive research, enhances clinical applications, enables cross-cultural comparisons, and contributes to a deeper understanding of social and interpersonal dynamics within the Portuguese context [1]. Validating the Fear-of-Intimacy Scale (FoIS) for the Portuguese population is important, despite its original development in 1991, because this scale was developed in a different cultural context [2]. Validation will ensure that it accurately measures fear of intimacy within the Portuguese cultural framework, which may differ in its perceptions of intimacy and interpersonal relationships [3]. Also, direct translations can introduce bias or alter meaning; validation will ensure that the scale retains its conceptual integrity and relevance after translation into Portuguese. Over time, scales may require re-evaluation to confirm that they remain reliable and valid [3]. For the Portuguese population, this process will ensure that the FoIS measures what it intends to, reflecting the population’s specific experiences [2]. While this scale was developed in 1991, intimacy-related issues remain relevant. Validation will ensure that the scale’s items are still applicable and resonate with contemporary relationship dynamics in Portugal. Having a validated version of the FoIS will enable researchers in Portugal to contribute to and compare findings in the broader international literature, fostering consistency in cross-cultural studies [3]. Ultimately, validation will ensure the scale’s relevance, accuracy, and cultural sensitivity, making it a reliable tool for clinical and research applications in Portugal [2].

Performing a multigroup analysis across individuals who are in relationships and those who are not is crucial for validating the Fear-of-Intimacy Scale for the Portuguese population [4]. People in relationships may experience fear of intimacy differently from those who are not, as interpersonal dynamics can vary. A multigroup analysis will ensure that the scale measures fear of intimacy effectively across both groups and will test for measurement invariance, confirming that the scale functions similarly for both [5]. Additionally, this analysis will help to assess whether cultural factors influence how fear of intimacy is experienced in different relationship contexts, ensuring the scale’s relevance and validity [4]. Understanding these differences can also inform more tailored interventions for individuals in or out of relationships.

Since the 1980s, societal shifts have delayed adult responsibilities, fostering a rise in casual relationships, particularly casual sex [6,7,8]. Engaging in these relationships is facilitated by freedom from long-term commitment [9,10], although risks such as susceptibility to sexually transmitted infections exist [11]. Casual sex has been linked to fear of intimacy [12,13], yet the intricacies of this relationship have not yet been fully grasped.

### 1.1. Fear of Intimacy

Initially emphasized by Erikson [14] as a key adult task, intimacy became associated with commitment [15] and psychological well-being [16,17]. Intimacy includes love, positive affection, commitment, reciprocity, and both physical and psychological closeness [18,19]. The fear of intimacy, as defined by Descutner and Thelen [20] (p. 219), refers to “an individual’s inhibited ability, due to anxiety, to exchange thoughts and feelings of personal significance with another individual who is highly valued”. Fear of intimacy is a multifaceted psychological construct involving anxiety or discomfort with closeness and vulnerability in relationships.

The fear of intimacy appears to be linked to risky sexual behavior, including frequent changes in sexual partners [12,21,22]. This fear is also associated with psychopathological conditions, particularly depression, especially among women [23,24]. Moreover, fear of intimacy is connected to emotional vulnerability (difficulties in exposed or expressing true feelings and emotions), known as alexithymia, and serves as a negative predictor for self-differentiation [25]. Also, fear of intimacy is related to fear of rejection or abandonment [26], trust issues, fear of dependence, negative self-image, past trauma or negative relationship experiences, fear of losing autonomy, avoidance of conflict, and fear of commitment. These themes often overlap, as fear of intimacy is rarely about one isolated factor.

Other studies [27,28] have shown past infidelity to be correlated with higher sociosexuality and lower commitment, suggesting a more permissive attitude towards infidelity. However, Rodrigues et al. [29] found that familiarity in casual sexual relationships may lead to higher levels of passion, commitment, and intimacy. This was influenced by factors like exclusivity, relationship objectives, and social/sexual activity frequency.

### 1.2. Sociosexual Orientation

Sociosexuality and casual sex are related concepts, but they are not the same. Sociosexuality is a personality trait or attitude and refers to an individual’s openness or willingness to engage in sexual relationships without requiring emotional closeness or commitment [30,31]. People with high sociosexuality are more likely to view sex as separate from emotional or romantic bonds. People with low sociosexuality prefer sex in committed, emotionally close relationships. Sociosexuality is studied on a continuum, and individuals can show variance within the lower (i.e., unrestricted sociosexual orientation) and higher (i.e., restricted sociosexual orientation) ends [32].

Casual sex is a behavior, and refers to the act of engaging in sexual activity outside of a committed or emotionally close relationship [33]. It typically includes one-night stands, friends with benefits, or other forms of sexual encounters without long-term commitment [34]. Unlike sociosexuality, casual sex describes a specific behavior, not a personality trait. People with low sociosexuality might occasionally engage in casual sex, but they might not feel comfortable or satisfied with it. Casual sex can occur for a variety of reasons (e.g., physical desire, experimentation, or cultural norms), and does not necessarily reflect a person’s overall attitude toward sex [34]. People with high sociosexuality are more likely to engage in casual sex, but engaging in casual sex does not necessarily mean that someone has high sociosexuality. Someone with low sociosexuality may engage in casual sex due to external factors (e.g., social pressure, curiosity), but may not derive much satisfaction from it [35].

Wongsomboom, Burleson, and Webster [36] (2020) found higher sociosexuality to be linked to increased sexual satisfaction and orgasm frequency in relationships. Simpson and Gangestad [30], as cited by Wei et al. [37], observed that those with unrestricted sociosexuality were more likely to initiate relationships early, consider multiple partners, and exhibit lower commitment and love. Individual differences in sociosexuality have been found to be associated with multiple aspects of infidelity in intimate relationships [38].

Alvarez et al. [33] highlighted emotional involvement, dating frequency, friendship level, and relationship termination as key dimensions of casual sexual relationships (CSRs). In addition, Garcia et al. [39] discovered that although affectionate behaviors were more commonly sought in romantic relationships than in casual sexual encounters, many participants, regardless of gender, still engaged in such behaviors during casual encounters. Studies on gender differences concerning casual sexual relationships (CSRs) yield conflicting results. Some suggest that men and those who are not in committed relationships are more inclined towards sociosexuality [27,40], while others argue that CSRs are common in both genders, driven by a desire for physical pleasure [8,41]. Schmitt [42] found that women tend to be more conservative in sociosexual behavior, though individual differences exist [43]. Grello et al. [44] observed increased depression symptoms in women post-casual sexual relationships. Wongsomboom, Burleson, and Webster [36] noted differences in orgasm and sexual satisfaction for women with lower sociosexuality in committed relationships versus casual sexual relationships (CSRs). Wesche, Claxton, and Waterman’s [45] systematic review found that CSR initiation can lead to emotional health issues, particularly in women. Negative effects are amplified by factors like alcohol consumption, sexual dissatisfaction, and partner unfamiliarity [45].

In many studies, sociosexual orientation is often operationalized through behaviors related to casual sex, especially when measuring the behavioral dimension of the construct. For instance, items assessing the frequency or history of casual sexual relationships are part of scales such as the Sociosexual Orientation Inventory (SOI).

Thus, using the terms synonymously can be a practical way to simplify communication in studies, particularly when the focus is on casual sexual behavior as an expression of high sociosexuality; this is the case in this study.

### 1.3. Relation Between Fear of Intimacy and Sociosexual Orientation

In the literature, both Fear of Intimacy and Sociosexual Orientation are important psychological constructs that influence interpersonal relationships and intimacy dynamics. In examining these relationships, the scales used to measure both constructs demonstrate strong convergent and divergent validity. The Fear-of-Intimacy Scale and the Sociosexual Orientation Inventory have both been validated as reliable measures within their respective domains. Convergent validity is supported by the positive correlations found between the Fear-of-Intimacy Scale and other measures of emotional closeness and relationship anxiety [20,46], as well as between the Sociosexual Orientation Inventory and measures of sexual behavior and attitudes toward relationships [30]. Divergent validity is also evident, as the scales distinguish between constructs that are not conceptually related, such as general personality traits or unrelated emotional experiences [30].

The relationship between fear of intimacy and engagement and sociosexual orientation is complex. For some individuals, fear of intimacy may lead them to engage in sociosexual orientation as a way to avoid the emotional vulnerability and closeness associated with more intimate relationships [47]. Sociosexual orientation as a coping mechanism allows the fulfillment of physical needs without the emotional investment or risk of intimacy [48,49]. Attachment theory suggests that individuals with insecure attachment styles, such as avoidant attachment, may be more likely to fear intimacy and seek out casual sexual encounters as a result [50,51]. These individuals may struggle with forming and maintaining close relationships, leading them to prefer casual and non-committal interactions [51,52]. Some people may use casual sex as a means to boost their self-esteem or seek validation from others [49]. Engaging in sexual encounters without emotional attachment may temporarily fulfill their need for validation or acceptance without risking the vulnerability associated with intimacy [53].

Societal norms, cultural beliefs, and peer influences can also play a role in shaping individuals’ attitudes and behaviors towards intimacy and casual sex. In cultures or social circles where casual sex is normalized or even encouraged, individuals may be more likely to engage in such behaviors, regardless of their fear of intimacy [54]. In contemporary times, the virtual realm, accessed through digital platforms, stands as a lasting consequence of the pandemic era, and has been identified as a key factor influencing shifts in interpersonal dynamics [55]. Simultaneously, the digitalization of relationships appears to facilitate casual, non-committal connections and a predisposition toward sociosexuality. Throughout the pandemic, there has been a notable surge in the use of technology for occasional virtual interactions and for introducing diversity and creativity into sexual experiences [56,57,58].

Fear of intimacy often stems from past experiences of rejection, abandonment, or trauma in close relationships. Some individuals may avoid intimacy altogether to protect themselves from potential emotional pain or disappointment, opting instead for casual and superficial connections [59]. Relationships, especially those of an intimate nature, now exhibit distinct characteristics. The meanings of intimacy and fidelity have undergone transformations, owing to the convenience with which relationships can be initiated or terminated at the mere click of a button [60].

The increasing significance of the physical aspect, particularly the sexual act, concerning intimacy, is evident in the findings of studies conducted by Muniruzzaman [61]. In a study focusing on young adults engaged in intimate relationships lasting less than a year, researchers [62] determined that women exhibited a higher degree of intimacy compared to men. This finding aligned with earlier study conclusions [63] suggesting that women tend to prioritize family relations and cultivate a heightened perception of intimacy within romantic relationships. In contrast, men appeared to be more oriented towards providing for the family, placing greater emphasis on the work environment.

### 1.4. The Relationship Between Fear of Intimacy and Sociosexual Orientation as a Public Health Issue

The relationship between Fear of Intimacy and Sociosexual Orientation has significant public health implications, as it affects mental health, sexual health, and relational well-being, which are core components of population health management [64].

Fear of Intimacy often stems from unresolved psychological issues, such as past trauma, attachment insecurities, or low self-esteem, which can lead to anxiety, depression, and emotional vulnerability [50]. Individuals with high sociosexual orientation (openness to casual sex without emotional closeness) may use these behaviors as coping mechanisms to avoid intimacy [49]. While these behaviors might offer temporary relief, they can exacerbate mental health challenges over time, creating a cycle of emotional instability and avoidance [16]. Public health strategies must address these patterns through mental health services that integrate relational counseling, reducing the stigma of seeking help for intimacy-related concerns [65].

Casual sexual behavior associated with unrestricted sociosexual orientation is linked to increased risk of sexually transmitted infections (STIs) and unintended pregnancies [11]. People with a fear of intimacy may engage in sociosexual behaviors without prioritizing safe sex practices, as their focus might be on avoiding emotional connection rather than fostering healthy relational dynamics [47]. Public health campaigns need to highlight the importance of safe sexual practices, particularly for individuals in non-committed relationships, while addressing the underlying emotional factors that drive these behaviors [41].

Fear of intimacy can lead to avoidance of long-term relationships or commitment, impacting family stability and the ability to form supportive social networks [10]. High sociosexual orientation in these individuals may further hinder the development of stable partnerships, which are protective factors for physical and mental health. Public health programs can focus on fostering relational skills and emotional resilience as a way to strengthen family systems and social bonds [38].

Avoidance of intimacy may increase feelings of loneliness and social isolation, both of which are linked to significant public health concerns like cardiovascular disease, weakened immune function, and mental health disorders [66]. Individuals engaging in sociosexual behaviors as a substitute for emotional connection may experience dissatisfaction or emotional disconnection, exacerbating social isolation. Interventions promoting social connectedness and emotional intimacy could mitigate these risks, enhancing overall community well-being [67].

Fear of intimacy and sociosexual orientation are influenced by societal norms, including those perpetuated by digital platforms [54]. The normalization of casual sexual relationships in digital spaces can exacerbate intimacy avoidance, creating new public health challenges around relational and sexual health [68]. Public health strategies must address the digitalization of relationships, promoting awareness of the psychological and health impacts of casual sexual behaviors in the absence of emotional connection [69].

Young adults and those with unresolved relational trauma may be particularly vulnerable to the interplay between fear of intimacy and sociosexual orientation, as they navigate emerging adulthood and identity formation [26]. Targeted education and intervention programs can provide these populations with the tools to foster healthy relational and sexual practices, reducing long-term public health burdens [38].

Drawing upon the existing body of literature and recognizing the scarcity of studies in this area, this research aims to adapt the Fear-of-Intimacy Scale for the Portuguese population and examine its association with sociosexual orientation, as measured by the Sociosexual Orientation Inventory. Based on previous validations of the Fear-of-Intimacy Scale [20,46,70,71,72,73,74,75,76], the hypothesis is raised that the Portuguese version of the Fear-of-Intimacy Scale will present three dimensions, just like most of the other validated versions.

## 2. Methods

### 2.1. Procedures

#### 2.1.1. Ethics

This study adhered to the ethical guidelines outlined in the Helsinki Declaration [77]. Approval for the research was obtained from the Scientific Council of the Universidade Católica Portuguesa. The survey included an informed consent document detailing the study’s objectives, voluntary participation, and the assurance of data anonymity and confidentiality. Only participants who provided their informed consent were included in the study.

#### 2.1.2. Transcultural Adaptation of the Fear-of-Intimacy Scale (FoIS)

The translation and back-translation of the English version of the Fear-of-Intimacy Scale (FoIS) was conducted in accordance with international guidelines [78] to ensure cross-cultural equivalence. Initially, two independent Portuguese translators, one of whom was a psychologist with expertise in the subject of the scale, carried out the translation from English to Portuguese. Subsequently, researchers and translators collaboratively assessed both versions to arrive at a consensus. Finally, an English translator reviewed a backward translation from Portuguese to English of the final version. The ultimate Portuguese version exhibited no significant changes in content compared to the original version. To ensure the accuracy of the items, an online survey that included the FoIS was administered to 25 participants, though these data were not included in the subsequent statistical analysis (Appendix A).

#### 2.1.3. Sampling

The participants were recruited between 1 September and 31 October 2024, using the snowball sampling method [79]. This involved reaching out to individuals from the general population through the distribution of the survey on various social media platforms, such as Facebook and Twitter. Inclusion criteria stipulated that participants needed to be of Portuguese nationality, native Portuguese speakers, and over 18 years old. Additionally, they were required to provide informed consent. Exclusion criteria encompassed not meeting the inclusion requirements or failing to complete the entire survey.

### 2.2. Participants

Initially, 732 questionnaires were collected. However, 59 of them were incomplete and, therefore, were excluded from the analysis. Consequently, the final sample consisted of 673 participants. Among these, 508 were women, constituting 75.5% of the sample, and their ages ranged from 18 to 73 years (M = 25.99; SD = 11.44). Regarding educational attainment, the majority of participants (52.5%) had completed 12 years of education, 8.6% had completed less than 12 years of education, and 38.9% were university students with a degree or more. Additionally, more than half of the participants (55.0%) reported being in a romantic relationship, while the remaining half were not.

### 2.3. Measures

The questionnaire comprised a section on sociodemographic information, covering variables such as sex assigned at birth, age, education, and romantic relationship status. Age and education were treated as continuous variables. To assess romantic status, participants were asked the question, “Are you currently in a romantic relationship?”, with the response options being “no” or “yes”.

#### 2.3.1. Fear-of-Intimacy Scale (FoIS)

Descutner and Thelen [20] developed the Fear-of-Intimacy Scale (FoIS) to gauge a “variable that influences intimacy (fear of intimacy) in a close relationship or at the prospect of a close relationship” (p. 218). Comprising 35 items, the scale employs a 5-point Likert-type rating system, ranging from 1 (not at all characteristic of me) to 5 (extremely characteristic of me), with nearly half of the items being reverse scored. Higher scores on the scale indicate greater levels of fear. The questionnaire is divided into two parts: the first requires participants to envision themselves in a close relationship, while the second instructs them to reflect on their past relationships.

Descutner and Thelen [20] identified three factors in their study, while Doi and Thelen [70] found two large factors, and Sherman and Thelen [76] found three factors. All three studies reported good reliability, with α = 0.93 for the total scale in Descutner and Thelen [20], α = 0.93 in Doi and Thelen [70], and α = 0.90 and α = 0.92 in Sherman and Thelen [76]. Subsequent adaptations and validations of the FoIS have been conducted, including versions for Chinese and American populations (α = 0.88 and α = 0.92, respectively, with three factors in both cases) by Ingersoll et al. [46]; versions for English and Mandarin speakers (α = 0.78 and α = 0.88, respectively, with three factors in both cases) by Ingersoll et al. [71]; and a version for Italian speakers (α = 0.91; α = 0.73; and α = 0.91, respectively, with two factors and a total score) by Senese et al. [75]. Various studies employing different samples have also been conducted, such as those involving emerging adults [73] and young adults [72]. Although there is no Portuguese version of the FoIS, Rohner et al. [74] incorporated a Portuguese sample in a multicultural study spanning 13 countries.

#### 2.3.2. Revised Sociosexual Orientation Inventory (SOI-R)

Building upon the original Sociosexual Orientation Inventory (SOI) introduced by Simpson and Gangestad in 1991 [30], Penke and Asendorpf [32] developed the Revised Sociosexual Orientation Inventory (SOI-R) in collaboration with researchers. Penke and Asendorpf [32] aimed to assess sociosexuality, capturing individual differences in people’s willingness to engage in uncommitted sexual relations. The SOI-R comprises nine items, with four drawn from the original SOI and five newly created for this version. It gauges three dimensions of sociosexuality: past behavior (quantifying the number and casualness of sexual partners), attitude toward sex without commitments, and sexual desire for individuals without any romantic involvement. Response options are provided on a Likert-type scale with nine choices, and the scoring varies for each dimension. Higher scores on the SOI-R suggest a non-restricted sociosexual orientation, indicative of more generalized promiscuous behavioral tendencies, while lower scores imply a restricted sociosexual orientation [32]. In terms of reliability, the Cronbach’s alpha values for males and females are consistent across the three factors (behavior, attitude, and desire) and the total scale. For example, in factor 1 (behavior), Cronbach’s alpha is 0.85 for males and 0.84 for females; in factor 2 (attitude), it is 0.87 for males and 0.83 for females; and in factor 3 (desire), it is 0.86 for males and 0.85 for females. The overall reliability of the total scale is robust, with a Cronbach’s alpha of 0.83 for males and females alike [32]. Regarding the Portuguese version by Neto [80], there were no structural or item-related changes. The reliability measures for sociosexual behavior (factor one) yielded a Cronbach’s alpha of 0.85, for sociosexual attitude (factor two), it was 0.70, and for sociosexual desire (factor three), it was 0.84. The global sociosexual index demonstrated strong internal consistency, with a Cronbach’s alpha of 0.87.

### 2.4. Data Analysis

The determination of the sample size was made in advance, taking into consideration the varying recommendations provided by structural equation modeling (SEM) experts. Kline [81] proposed a guideline suggesting a ratio of 20 observations to the number of free model parameters (*N*:*q*), while Schreiber et al. [82] recommended a ratio of 10 to 1. Considering that the instrument under validation in this study comprises 35 items, Kline’s [81] guideline would necessitate 700 participants, and Schreiber et al.’s [82] recommendation would require 350 participants. Opting to adhere to the more stringent criterion, as suggested by Kline [81], a sample of 673 participants was secured. The sample’s power, calculated using Giga Calculator (https://www.gigacalculator.com, accessed on 10 November 2024), was determined to be 0.9963, corresponding to 99.63% [83].

The evaluation of item normality encompassed the examination of skewness (SI < 3) and kurtosis (KI < 10), as recommended by Kline [81]. To assess multicollinearity, tolerance (>0.100) and variance inflation factor (VIF) (<10) were examined, with a particular focus on detecting correlations exceeding 0.80 [84]. Additionally, a comprehensive assessment of items was conducted, including descriptives, scale mean if the item is deleted, scale variance if the item is deleted, corrected total item correlation, and Cronbach’s alpha if the item is deleted.

Exploratory factor analysis (EFA) was conducted to identify the underlying factor structure of the dataset and explore the relationships between observed variables. This technique is particularly useful when the goal is to discover patterns or groups of variables that share a common underlying factor, without a predetermined hypothesis about the number or nature of the factors. EFA allowed us to reduce the data into a smaller set of uncorrelated factors, facilitating a more interpretable understanding of the data. The analysis involved examining the factor loadings, eigenvalues, and variance explained to determine the most appropriate number of factors. Additionally, the Kaiser–Meyer–Olkin (KMO) measure and Bartlett’s Test of Sphericity were employed to assess the suitability of the data for factor analysis.

To assess the adequacy of the instruments, confirmatory factor analyses (CFAs) were conducted on the Portuguese sample to evaluate the goodness of fit using the Diagonal Weighted Least Squares (DWLS) estimation method. This method is particularly suited to ordinal data, as it accounts for non-normality and provides robust parameter estimates. Various fit indices were considered for the CFA models, including the root mean square error of approximation (RMSEA), the comparative fit index (CFI), the incremental fit index (IFI), the Tucker–Lewis index (TLI), the goodness of fit index (GFI), and the standardized root mean square residual (SRMR). Following the criteria established by Hu and Bentler [85], a very good model fit was identified when the CFI, IFI, TLI, and GFI were ≥0.95, the RMSEA was ≤0.05, and the SRMR was ≤0.05. For an acceptable model fit, Hooper et al. [86] suggest values of ≥ 0.90 for the CFI and IFI, ≤0.08 for the RMSEA, and ≤0.10 for the SRMR. Additional reporting includes the Satorra–Bentler chi-square (χ^2^), general model significance (*p*), and relative chi-square (χ^2^/df) [87].

Although the Sociosexual Orientation Inventory (SOI) has been validated for the Portuguese population, conducting a confirmatory factor analysis (CFA) remains essential. Each sample presents unique demographic, cultural, and contextual characteristics, and CFA ensures that the previously established factor structure—such as the division into attitudes, behaviors, and desires—is replicable in the new sample. The validity and reliability of an instrument can vary across samples, even within the same country. A CFA evaluates whether the relationships between items and factors remain consistent with the theoretical framework and prior research findings. Subtle differences in culture, generational norms, or even the translation and adaptation process can influence the model’s fit. Importantly, the CFA provides a detailed assessment of the proposed factorial model by examining overall model fit (how well the observed data align with the theoretical structure), factor loadings (the strength of associations between items and their corresponding factors), and factor independence (whether the factors are distinct from one another). A model that performs well in one population may require adjustments when applied to another, highlighting the need for local adaptations [87].

Multigroup confirmatory factor analyses (CFAs) were employed to assess the validity of the factor structure of the scales concerning individuals in or not in a romantic relationship. Multigroup CFAs are essential for verifying that the Fear-of-Intimacy Scale reliably and validly assesses the construct across individuals with different relational experiences. This step is critical for ensuring the scale’s accuracy, theoretical integrity, and applicability to diverse populations [4].

Four levels of measurement invariance were examined: configural, metric, scalar, and error variance invariance. The evaluation of progressively constrained models was conducted through the comparison of pairs of nested models (∆) using the root mean square error of approximation (RMSEA), comparative fit index (CFI), and standardized root mean square residual (SRMR). A change of ≥0.01 in the CFI, ≥0.015 in the RMSEA, and ≥0.03 in the SRMR indicates a statistically significant decrease in the model fit [5].

Pearson correlations were computed for continuous variables, while Spearman correlations were employed when variables were ordinal or nominal. The interpretation of correlations followed Cohen et al.’s [88] guidelines, categorizing correlations as weak (between 0 and 0.3), moderate (between 0.3 and 0.5), strong (between 0.5 and 0.7), and very strong (between 0.7 and 1), and either positive or negative.

For evaluating model reliability and convergent and discriminant validity, a range of statistical measures were employed. These included Cronbach’s alpha coefficients, MacDonald’s omega, composite reliability (CR, where a value of 0.70 or higher suggests good model reliability), the average variance extracted (AVE, with a threshold of 0.50 or higher indicating adequate convergence), and the square root of the AVE (which should be higher than the highest correlation with any other latent variable) [89].

The significance threshold for statistical analysis was predetermined at 0.05. The analytical procedures were conducted using SPSS version 28, and JAMOVI version 2.3.28.

## 3. Results

### 3.1. Preliminary Analyses

The items of the Fear-of-Intimacy Scale demonstrate skewness and kurtosis scores within the acceptable range, indicating a normal distribution. Additionally, the tolerance and variance inflation factor (VIF) fall within the reference values, confirming the absence of multicollinearity. Moreover, the Cronbach’s alpha for the total scale remains consistent even when individual items are removed, with the exception of a marginal improvement observed for item 31 (Table 1).

### 3.2. Validation of the Fear-of-Intimacy Scale for the Portuguese Population

#### 3.2.1. Exploratory Factor Analysis

An exploratory factor analysis (EFA) was conducted to examine the underlying factor structure of the 35-item Fear-of-Intimacy Scale using Principal Axis Factoring (PAF) with Varimax rotation, which is appropriate for identifying orthogonal (uncorrelated) factors. Before conducting the EFA, the Kaiser–Meyer–Olkin (KMO) measure of sampling adequacy and Bartlett’s Test of Sphericity were performed to assess the suitability of the data for factor analysis. The KMO value was 0.89, which is above the recommended threshold of 0.6, indicating that the sample size was adequate for factor analysis. Bartlett’s Test of Sphericity was statistically significant (χ^2^(66) = 1453.25, *p* < 0.001), confirming that correlations between items were sufficiently large to proceed with factor analysis. The determinant of the correlation matrix was calculated as 0.000053, which is greater than the threshold of 0.00001, suggesting that multicollinearity was not a concern and that the data were appropriate for factor analysis. The anti-image correlation matrix was examined to assess the adequacy of the individual items for factor analysis. The values in the anti-image matrix ranged from 0.67 to 0.92, well above the recommended minimum value of 0.5, indicating that each item had adequate correlations with other items and contributed meaningfully to the factor structure. Additionally, the correlations between items ranged from 0.30 to 0.75, showing that the inter-item correlations were appropriate for factor analysis. None of the correlations were so low as to suggest redundancy, or so high as to indicate multicollinearity issues. Using the Kaiser criterion (eigenvalues > 1) and a scree plot, three factors were retained, accounting for 71.5% of the total variance. The eigenvalues for the three factors were 3.46, 2.34, and 1.21, respectively. The factor loadings after Varimax rotation ranged from 0.42 to 0.89, indicating strong associations between items and their respective factors. The rotated solution using Varimax rotation resulted in three interpretable factors: imagined fear of closeness, imagined openness, and past fear closeness.

#### 3.2.2. Confirmatory Factor Analysis

Descutner and Thelen [20], the authors of the Fear-of-Intimacy Scale (FoIS), proposed that fear of intimacy encompasses three underlying factors, but maintained that the scale operates as a unidimensional construct. However, previous studies have reported mixed findings regarding whether the FoIS truly reflects a single-factor structure. Given this uncertainty and the absence of validation evidence for the scale within Portuguese populations, we decided to conduct a confirmatory factor analysis (CFA) to evaluate its factor structure and ensure cultural consistency. As highlighted in the previously discussed studies, the 35 items can be grouped into one, two, or three distinct underlying components. Our EFA resulted in three factors.

**Figure 1:**
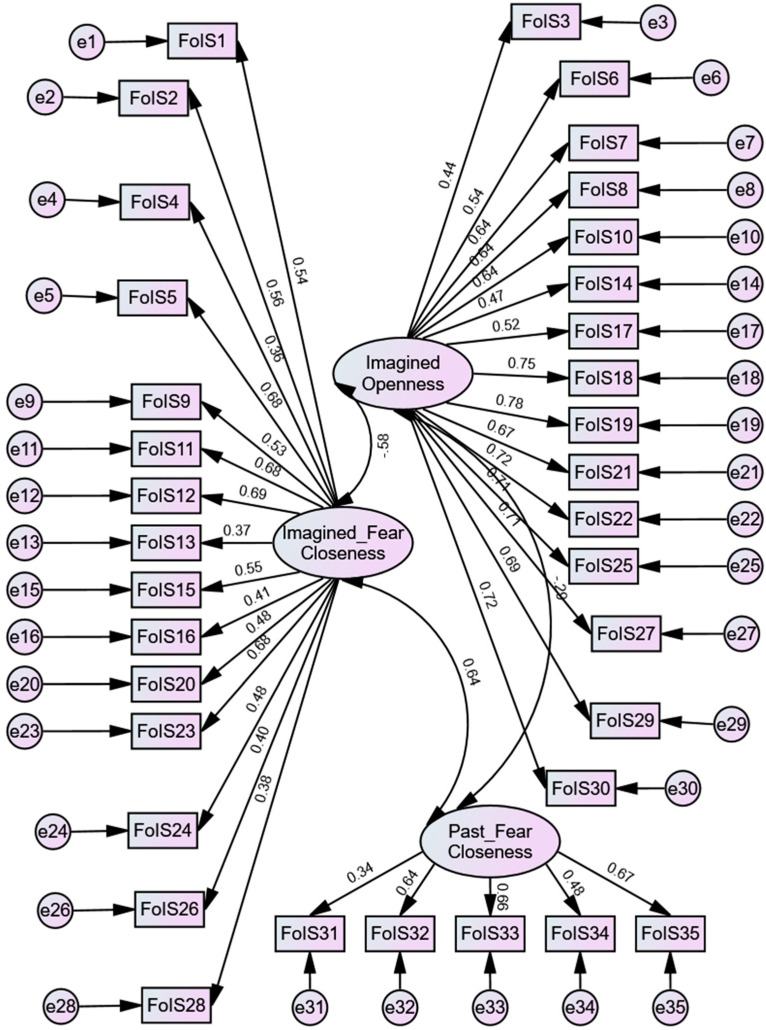
The final CFA model of the FoIS.

The confirmatory factor analysis results also show that the model with the best fit is the one that includes the three factors previously mentioned (Figure 1, Table 2).

#### 3.2.3. Measurement of Invariance

The results regarding the measurement of invariance of the FoIS across individuals being in or not in a romantic relationship are detailed in Table 3. Configural invariance concerning being in or not in a romantic relationship was substantiated during the initial step of the multigroup confirmatory factor analyses (CFAs). This pattern persisted with metric and scalar invariance. However, attaining invariance in error variance proved unfeasible, as the comparison of the comparative fit index (CFI) between error variance invariance and metric invariance exceeded the established reference values. Nevertheless, these findings provide support for the invariance of the FoIS concerning being in a romantic relationship or not (Table 3).

#### 3.2.4. Model Reliability

Reliability measures for the factors of the FoIS are outlined in Table 4. Positive and statistically significant correlations were observed among all dimensions of the FoIS. Noteworthy is the absence of significant differences between Cronbach’s alpha (α) and McDonald’s omega (ω). Both Cronbach’s alpha and McDonald’s omega values fall within acceptable limits, affirming the reliability of the FoIS as a measure. Additionally, the composite reliability, average variance extracted (AVE), square root of AVE, mean, and standard deviation were computed (Table 4), with all values falling within the recommended range.

### 3.3. Assessing the Revised Sociosexual Orientation Inventory (SOI-R)

#### 3.3.1. Confirmatory Factor Analysis

**Figure 2:**
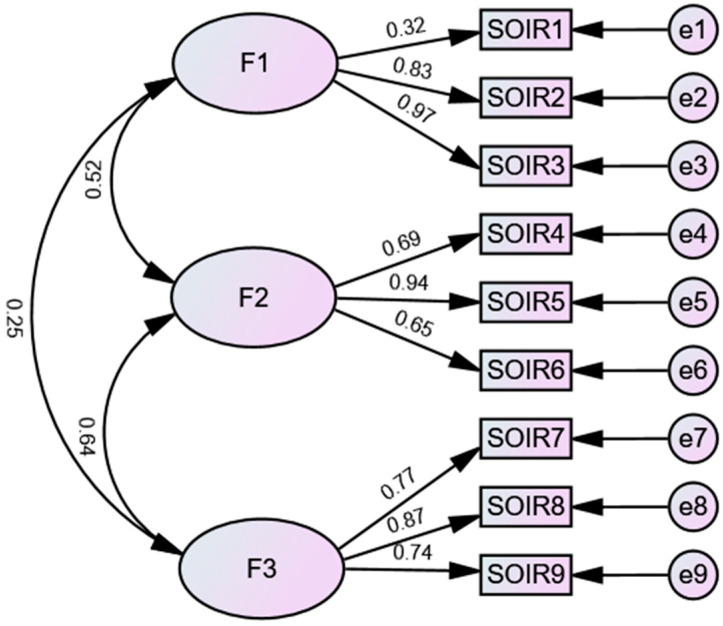
The final CFA model of the SOI-R.

The compatibility of the SOI-R with the sample is highly satisfactory [χ^2^(24) = 2.666; CFI = 0.985; TLI = 0.978; IFI = 0.985; GFI = 0.980; RMSEA = 0.050 (CI 0.035–0.060); SRMR = 0.035; AIC = 105.988] (Figure 2).

#### 3.3.2. Model Reliability

Table 5 provides reliability indices for the factors of the SOI-R. Positive and statistically significant correlations can be observed across all dimensions of the SOI-R. Notably, some distinctions are noted between Cronbach’s alpha (α) and McDonald’s omega (ω). Overall, the SOI-R emerges as a reliable measurement tool. Additionally, the composite reliability, average variance extracted (AVE), square root of AVE, mean, and standard deviation were computed (Table 6), with all values falling within the accepted range.

### 3.4. Relation Between FoIS and SOI-R

The overall Fear-of-Intimacy Scale (FoIS) shows positive and significant correlations with both the total score and the Sociosexual Desire subscale of the SOI-R. Imagined Fear of Closeness is positively and significantly correlated with all SOI-R dimensions, except for Sociosexual Behavior, which displays a negative correlation. In contrast, Past Fear of Closeness is positively and significantly correlated with all SOI-R dimensions. Imagined Openness does not correlate with any SOI-R dimensions. However, it is important to highlight that the strength of these correlations is relatively low, as shown in Table 6.

## 4. Discussion

Adapting the Fear-of-Intimacy Scale (FoIS) for the Portuguese population is essential for culturally sensitive research, clinical practice, and cross-cultural comparisons [1]. Although developed in 1991, the FoIS needs validation to ensure that it accurately reflects Portugal’s unique views on intimacy and relationships, as cultural differences can affect its applicability [2,3]. Direct translations may introduce biases, making validation crucial for maintaining its integrity. Additionally, societal changes and evolving relationship dynamics require periodic re-evaluation to ensure that the scale remains reliable and relevant [3]. In Portugal, where traditional and modern influences shape relationships, a validated FoIS supports both international research contributions and enhances its use in clinical settings [2]. Understanding fear of intimacy is vital for addressing psychological well-being and relational challenges within the sociocultural context [16,17].

The original Fear-of-Intimacy Scale (FoIS) by Descutner and Thelen [20] identified a primary factor explaining 33.4% of variance, with secondary factors accounting for 7.4% and 5.2%, but lacked details on item–factor associations. Subsequent studies revealed variations in factor structure: Sherman and Thelen [76] reported three factors, while Doi and Thelen [70] and the Italian version [75] identified two. In contrast, Ingersoll et al. [46,71] consistently found three factors across Chinese, American, and bilingual samples, and the Portuguese adaptation also supports a three-factor structure, demonstrating excellent model fit and strong psychometric properties. Despite general support for a three-factor model, cultural nuances may influence factor grouping, as seen in the Italian adaptation [75]. Reliability is typically strong (α ≥ 0.88) across most versions, though some exceptions exist, such as the English version in 2012 (α = 0.78) [71] and the Italian subscale (α = 0.73) [75]. Cross-cultural studies highlight the scale’s adaptability, though translations may impact reliability, as observed in differences between the Mandarin [71] and Chinese [46] versions. Additionally, most adaptations abandon the original two-part structure (envisioning vs. reflecting) in favor of a unified format.

Fear of intimacy, as defined by Descutner and Thelen [20], involves discomfort and anxiety about emotional closeness and vulnerability. It is linked to conditions like depression, alexithymia, and relational insecurities such as fear of rejection or commitment [23,26]. These issues often intersect with trust, trauma, and avoidance behaviors. Our findings reveal a link between intimacy fears and sociosexual tendencies, with casual sexual encounters fulfilling needs while avoiding vulnerability. Also, researchers connect fear of intimacy to risky sexual behaviors, like frequent partner changes [21]. Rodrigues et al. [29] note that exclusivity and activity levels influence intimacy, passion, and commitment, even in casual relationships.

The interplay between fear of intimacy and sociosexual orientation reflects a psychological mechanism whereby individuals with intimacy-related anxieties may gravitate toward sociosexual behaviors to avoid emotional vulnerability [47]. For some, casual sexual encounters provide a way to meet physical needs without the emotional risks of deeper connections [48,49]. Attachment theory underscores this association, as individuals with avoidant attachment styles often fear intimacy and favor non-committal interactions [50,52].

Empirical findings reveal positive correlations between the Fear-of-Intimacy Scale (FoIS) and the Revised Sociosexual Orientation Inventory (SOI-R), particularly its desire subscale, suggesting that individuals with intimacy fears may compensate by engaging cognitively with relational fantasies or sociosexual scenarios without acting on them behaviorally [12,30]. This aligns with theories that such individuals manage relational anxieties by substituting emotional intimacy with physical or imagined interactions, thus maintaining relational engagement while avoiding vulnerability [31].

Despite the generally weak correlations observed, there is a predominantly positive and significant association between the Fear-of-Intimacy Scale (FoIS) and the Revised Sociosexual Orientation Inventory (SOI-R), along with their respective subscales. This aligns with the findings of Traeen [12], supporting the notion that individuals exhibiting a sociosexual orientation, indicative of heightened sexual interest in casual partners, also tend to experience greater fear of intimacy. The sexual desire subscale of the SOI-R demonstrates noteworthy correlations with various FoIS subscales. The study emphasizes that individuals that are inclined towards casual relationships may concurrently experience apprehensions associated with security, trust, commitment, and intimacy [90]. This observation suggests a potential fear of attachment among these individuals. Those with high sociosexuality often dissociate sex from emotional bonds, while those with low sociosexuality prefer sex within committed, emotionally intimate relationships [32]. Nevertheless, the outcomes presented here are at odds with the findings reported by Ka et al. [91], which assert that sociosexual behavior is a predictor of attitudes toward consensual non-monogamy (CNM) relationships. According to their research, this association became more pronounced when individuals exhibited higher levels of secure attachment.

While our findings indicate a tendency for individuals that are fearful of establishing stable relationships characterized by trust, security, commitment, and intimacy to potentially compensate by investing in the sexual aspects of casual relationships, aligning with the conclusions of Simpson and Gangestad [30], who previously identified that individuals with a sociosexual orientation tend to engage in relationships with lower levels of personal investment, commitment, and love, these results contradict those of Ka et al. [91]. These findings also align with those of Ribeiro et al. [92], revealing that avoiding romantic attachment serves as a positive predictor of intimacy, contrary to the existing literature.

The positive and significant correlations between the overall Fear-of-Intimacy Scale (FoIS) and both the total score and the Sociosexual Desire subscale of the SOI-R can be understood in light of the underlying constructs measured by these scales. These findings are consistent with the conclusions drawn by Greszta et al. [50], which suggest that individuals in both short-term and long-term relationships exhibit a higher intensity of secure attachment style and increased levels of intimacy and commitment. In contrast, those engaged in friend-with-benefits relationships demonstrated a higher intensity of the avoidant attachment style and a non-restrictive sociosexual orientation. In fact, some studies have found significant associations between high sociosexuality and sexual satisfaction, lower levels of commitment, and relational dynamics emphasizing physical over emotional connections [30,36]. Additionally, higher sociosexuality has been linked to infidelity and less stable romantic commitments [38].

The FoIS primarily assesses discomfort or anxiety related to emotional closeness, while the Sociosexual Desire subscale reflects the intensity of sexual thoughts and fantasies about casual sex. Both constructs may intersect through a heightened focus on interpersonal dynamics and relational concerns. Individuals scoring high on fear of intimacy might simultaneously exhibit a preoccupation with relationships, which could manifest in higher sociosexual desire [66]. Sociosexual orientation reflects an individual’s openness to engaging in sexual relationships without emotional closeness or commitment [30,31].

Those with a fear of intimacy may experience relational ambivalence—wanting connection but also fearing it [66]. Sociosexual desire may serve as an outlet for relational desires that avoids the emotional vulnerability associated with deeper connections [93]. Despite fearing intimacy, individuals might still experience significant sexual or relational drives, as measured by sociosexual desire. These drives might exist independently of their discomfort with emotional closeness, creating the observed positive correlation. Some individuals may compensate for their fear of intimacy by engaging in more imaginative or indirect relational behaviors, such as fantasizing or thinking about casual sexual encounters, which are captured by the Sociosexual Desire subscale [94].

The positive correlations with Sociosexual Desire and Attitudes, but the negative correlation with Sociosexual Behavior, reveal a conflict between cognitive openness to casual relationships and behavioral avoidance due to fears of vulnerability [95]. This pattern is characteristic of individuals high in Imagined Fear of Closeness, reflecting discomfort with emotional intimacy even in hypothetical scenarios [94].

While Sociosexual Desire and Attitudes suggest openness to casual sex and its associated fantasies, such individuals may perceive these as less risky than committed relationships. However, their avoidance of actual sociosexual behavior highlights the protective strategies they employ to avoid emotional or physical vulnerability [95]. Sociosexual Behavior reflects actual engagement in casual sexual activities. The negative correlation with Imagined Fear of Closeness indicates that individuals with high fear of closeness are less likely to act on sociosexual tendencies [96]. This may stem from their avoidance of both emotional and physical intimacy, as physical encounters carry potential risks of vulnerability [94]. Such individuals might fantasize about sociosexual scenarios (evidenced by positive correlations with desire and attitudes), but refrain from acting on them due to anxiety, fear, or a desire to avoid complications [96]. Their avoidance of real-world interactions suggests that they engage cognitively or attitudinally with sociosexuality, while avoiding behaviors that feel emotionally risky.

Research by Hudson and Fraley [97] supports this dynamic: individuals with high attachment anxiety often seek sexual closeness to alleviate insecurity, while those high in attachment avoidance use casual sex for social validation, but avoid deeper connection. Thus, imagined scenarios may feel safer, as they lack the vulnerability inherent in real-world behavior. Positive correlations between Past Fear of Closeness and all dimensions of sociosexual orientation (SOI-R) suggest that past relational difficulties may drive greater sociosexual openness as a way to reconcile intimacy fears with relational needs [41]. Past Fear of Closeness reflects anxiety around emotional intimacy rooted in unresolved trust or vulnerability issues, shaping future relational dynamics [66,90]. Individuals with such fears may express heightened sociosexual desires, finding that fantasies or casual encounters are a way to engage relationally without risking emotional intimacy [93]. Similarly, permissive attitudes and behaviors toward casual sex can provide a coping mechanism, reframing intimacy as physical rather than emotional [98].

Unlike Imagined Fear of Closeness, which relates to hypothetical scenarios, Past Fear of Closeness involves real relational challenges. Casual sexual behaviors may help to fulfill relational needs while maintaining emotional distance, offering a less vulnerable way to relate to others [99]. Attachment insecurities or ambivalence from these fears often align with sociosexual openness as a compensatory strategy for managing intimacy-related anxieties [98,99].

### 4.1. Public Health Implications

The intersection of fear of intimacy and sociosexual orientation presents significant challenges for mental, relational, and sexual health, which are integral to public health strategies [64]. Fearful individuals engaging in sociosexual behaviors may neglect safe sexual practices, increasing risks of sexually transmitted infections (STIs) and unintended pregnancies. Public health campaigns must address these behaviors by promoting safe sex practices while considering the underlying psychological factors driving them [36,90]. Additionally, fear of intimacy can lead to relational instability, social isolation, and mental health challenges, such as depression and anxiety. These outcomes underscore the need for programs fostering relational skills, emotional resilience, and social connectedness to mitigate these risks [16,66]. Addressing these issues is particularly critical for young adults and individuals with unresolved relational trauma, who are especially vulnerable to intimacy avoidance and its consequences.

The digitalization of relationships and the normalization of casual sex through online platforms further complicate this dynamic. Virtual spaces often reinforce intimacy avoidance by promoting transient connections over meaningful relationships [69]. Public health initiatives must educate individuals about the relational and psychological impacts of these trends, encouraging healthier digital and relational practices [95,98].

### 4.2. Limitations and Future Research

This study has certain limitations that warrant consideration. Firstly, the scarcity of research on the desire for casual sex and fear of intimacy within the Portuguese population presents a challenge in contextualizing the findings. However, this scarcity also positions the study as original, contributing unique insights.

One limitation of this study is the wide age range of participants (18–73 years), which could introduce variability in behaviors and thoughts influenced by age-related factors. Although the mean age of the sample was 25.99 years, and most participants were within a relatively narrow age group, we did not include age as a key variable in the statistical analysis. As a result, we were unable to control for potential age-related differences in our findings. Future studies may benefit from a more homogeneous age group, or include age as a moderating variable to further investigate its influence on the outcomes observed in this study.

Methodological limitations are also noteworthy. The non-representativeness of the Portuguese population urges caution in generalizing the results. The inability to replicate the factorial structure of the original Fear-of-Intimacy Scale (FoIS) version underscores the necessity for further investigations in this specific population.

A longitudinal approach, rather than a cross-sectional one, would provide a more comprehensive understanding of the implications of casual sex for mental and physical health over time. This could yield more precise intervention recommendations.

Additionally, exploring other variables, such as pornography consumption and engagement in cybersex, could enhance our understanding of sexual behavior and attitudes. These phenomena, which are witnessing a significant surge, have been linked to the central constructs of this study—occasional sexual relations and intimacy. Finally, future research could explore the interplay of occasional sexual relations and intimacy in conjunction with variables like sexual orientation and within diverse cultural contexts, offering a more nuanced perspective on these dynamics.

## 5. Conclusions

The Portuguese adaptation of the Fear-of-Intimacy Scale (FoIS) is crucial for understanding intimacy-related anxieties within Portugal’s cultural and relational context. The scale’s validation and reliability allow for culturally sensitive research and clinical applications, especially given the evolving dynamics of relationships. Fear of intimacy is closely linked to relational insecurities, trust issues, and avoidant behaviors, often manifesting in a preference for sociosexual behaviors over emotionally vulnerable connections. This connection between fear of intimacy and sociosexual orientation highlights a psychological mechanism whereby individuals manage relational anxieties through casual sexual fantasies or behaviors, avoiding deeper emotional risks.

Empirical findings suggest that individuals with high fear of intimacy exhibit positive correlations with sociosexual desire and attitudes, but avoid sociosexual behaviors. This reflects a cognitive openness to casual relationships, alongside the employment of protective strategies against vulnerability. Attachment theories provide further insights, showing how past relational fears and avoidant tendencies shape these patterns, emphasizing the role of casual sexual behaviors as a coping mechanism for managing intimacy fears.

The interplay between intimacy avoidance and sociosexual tendencies has significant public health implications. Fearful individuals may neglect safe sexual practices or experience relational instability and mental health challenges, underscoring the need for public health initiatives focused on emotional resilience, relational skills, and safe sexual behaviors. The growing role of digital platforms in promoting transient connections intensifies these challenges, calling for education on healthier relational and digital practices. Addressing these issues holistically can mitigate risks and promote well-being in vulnerable populations.

## Figures and Tables

**Table 1:** Descriptive statistics of the Fear-of-Intimacy Scale (FoIS) items.

	M	SD	σ^2^	Sk (SD 0.09)	β2 (SD 0.19)	±	VIF	Scale Mean If Item Deleted	Scale Variance If Item Deleted	Corrected Total Item Correlation	α If Item Deleted
Total	2.31	0.59	0.35	0.16	−0.62						0.908 (total)
FoIS1	2.32	1.21	1.46	0.58	−0.71	0.59	1.69	78.65	404.46	0.45	0.906
FoIS2	2.58	1.27	1.61	0.47	−0.84	0.61	1.64	78.58	402.08	0.46	0.905
FoIS3	1.78	1.05	1.09	1.17	0.38	0.72	1.38	78.31	404.77	0.38	0.907
FoIS4	2.32	1.19	1.42	0.57	−0.63	0.89	1.24	79.11	411.30	0.31	0.907
FoIS5	1.93	1.21	1.46	1.30	0.68	0.53	1.90	78.57	395.36	0.61	0.903
FoIS6	2.32	1.13	1.27	0.67	−0.26	0.64	1.56	78.96	402.75	0.44	0.906
FoIS7	1.95	1.02	1.04	1.08	0.75	0.50	2.01	78.58	400.36	0.53	0.904
FoIS8	2.23	1.29	1.67	0.69	−0.72	0.54	1.86	78.94	405.54	0.46	0.905
FoIS9	2.48	1.16	1.34	0.44	−0.66	0.63	1.60	78.66	401.10	0.44	0.906
FoIS10	2.43	1.28	1.65	0.43	−0.96	0.54	1.86	78.42	399.42	0.54	0.904
FoIS11	2.13	1.14	1.30	0.81	−0.15	0.542	1.85	78.47	395.37	0.56	0.904
FoIS12	2.63	1.33	1.77	0.31	−1.10	0.53	1.87	78.76	396.38	0.62	0.903
FoIS13	2.61	1.32	1.74	0.39	−1.03	0.80	1.25	78.27	408.97	0.28	0.908
FoIS14	2.76	1.34	1.78	0.23	−1.10	0.73	1.37	78.29	405.66	0.34	0.907
FoIS15	3.02	1.33	1.78	0.03	−1.13	0.63	1.59	78.14	399.70	0.45	0.906
FoIS16	2.63	1.34	1.79	0.33	−1.07	0.77	1.30	77.87	406.53	0.32	0.908
FoIS17	2.42	1.20	1.45	0.53	−0.62	0.66	1.52	78.26	401.04	0.43	0.906
FoIS18	2.52	1.18	1.38	0.39	−0.73	0.38	2.65	78.48	395.55	0.60	0.903
FoIS19	1.62	0.94	0.89	1.50	1.53	0.32	3.16	78.37	392.71	0.68	0.902
FoIS20	2.48	1.16	1.35	0.40	−0.73	0.66	1.58	79.28	407.98	0.44	0.906
FoIS21	1.88	0.99	0.98	1.00	0.37	0.49	2.03	78.42	400.10	0.52	0.905
FoIS22	2.14	1.11	1.22	0.70	−0.34	0.45	2.24	79.01	402.61	0.56	0.904
FoIS23	2.85	1.28	1.63	0.15	−1.05	0.54	1.87	78.75	397.44	0.61	0.903
FoIS24	2.52	1.09	1.19	0.28	−0.62	0.67	1.50	78.05	405.55	0.36	0.907
FoIS25	2.48	1.26	1.59	0.44	−0.80	0.46	2.16	78.37	399.46	0.58	0.904
FoIS26	1.76	0.98	0.95	1.36	1.42	0.75	1.34	78.42	406.99	0.34	0.907
FoIS27	1.37	0.82	0.67	2.40	5.45	0.43	2.32	79.14	403.70	0.54	0.905
FoIS28	1.89	1.08	1.16	1.19	0.70	0.67	1.49	79.52	413.89	0.33	0.907
FoIS29	1.86	1.01	1.02	1.12	0.67	0.44	2.26	79.01	399.74	0.58	0.904
FoIS30	2.70	1.40	1.95	0.20	−1.24	0.44	2.28	79.04	401.92	0.56	0.904
FoIS31	2.69	1.36	1.85	0.19	−1.19	0.84	1.19	78.19	415.38	0.15	0.911
FoIS32	2.27	1.30	1.70	0.63	−0.80	0.63	1.60	78.20	401.35	0.41	0.906
FoIS33	2.77	1.39	1.92	0.20	−1.22	0.59	1.69	78.63	400.51	0.45	0.906
FoIS34	2.34	1.39	1.93	0.62	−0.94	0.74	1.35	78.12	411.48	0.22	0.909
FoIS35	2.25	1.10	1.20	0.53	−0.53	0.64	1.57	78.55	400.23	0.42	0.906

M = mean; SD = standard deviation; σ^2^ = variance; Sk = skewness; β2 = kurtosis; ± = tolerance; VIF = variance inflation factor; α = Cronbach’s alpha.

**Table 2:** Goodness of fit indexes for the models assessed for the Portuguese version of the FoIS.

										RMSEA CI 90%	
	χ^2^	DF	χ^2^/DF	IFI	TLI	CFI	GFI	SRMR	RMSEA	LO90	HI90
One factor, eight error covariances	2752	552	4.99	0.907	0.900	0.907	0.979	0.080	0.077	0.074	0.080
Two factors, two error covariances	2732	557	4.90	0.908	0.902	0.908	0.979	0.080	0.076	0.073	0.079
Three factors	1092	557	1.96	0.977	0.976	0.977	0.992	0.053	0.038	0.034	0.041

Note. χ^2^ = chi-squared; DF = default freedom; IFI = incremental fit index; TLI = Tucker–Lewis index; CFI = comparative fit index; GFI = SRMS = standard root mean square; RMSEA = root mean square error of approximation; CI = confidence interval.

**Table 3:** Multigroup CFAs of the FoIS according to being in or not being in a romantic relationship.

	χ^2^	df	χ^2^/df	RMSEA (CI)	CFI	IFI	SRMR	Comparisons	Δ RMSEA	Δ CFI	Δ SRMR
Configural invariance	1095	550	1.992	0.038 (0.035–0.042)	0.910	0.911	0.055	NA	NA	NA	NA
Metric invariance	1124	571	1.969	0.038 (0.035–0.041)	0.908	0.91	0.055	Configural vs. metric	0.000	0.002	0.000
Scalar invariance	1136	586	1.939	0.037 (0.034–0.041)	0.909	0.91	0.057	Metric vs. Scalar	0.001	0.001	0.002
Error variance invariance	1316	622	2.116	0.041 (0.038–0.044)	0.885	0.886	0.064	Scalar vs. error variance	0.004	0.024	0.007

Note. χ^2^ = chi-squared; df = degrees of freedom; IFI = incremental fit index; CFI = comparative fit index; RMSEA = root mean square error of approximation; CI = confidence interval; SRMS = standard root mean square; ∆ RMSEA = change in RMSEA compared with previous model (expressed in absolute values); ∆ CFI = change in CFI compared with previous model (expressed in absolute values); ∆ SRMR = change in SRMR compared with previous model (expressed in absolute values). All models are significant at *p* < 0.001.

**Table 4:** Correlations, Cronbach’s alpha, McDonald’s omega, composite reliability, average variance extracted (AVE), AVE square roots, and mean and standard deviation of FoIS.

				Pearson’s Correlations					
	0	1	2	3	α	ω	CR	AVE	Mean (*SD*)
0. Total	**0.715**				0.89	0.89	0.910	0.511	2.31 (0.59)
1. Factor 1	0.852 **	**0.708**			0.85	0.85	0.891	0.501	2.29 (0.67)
2. Factor 2	0.825 **	0.463 **	**0.776**		0.91	0.91	0.925	0.602	2.25 (0.76)
3. Factor 3	0.577 **	0.493 **	0.209 **	**0.758**	0.70	0.70	0.852	0.574	2.55 (0.91)

Note: ** *p* < 0.001; α = Cronbach’s alpha; ω = McDonald’s omega; CR = composite reliability; AVE = average variance extracted; **bold** (diagonal) = AVE square roots; *SD* = standard deviation; McDonald’s omega cannot be calculated because number of items is less than 3.

**Table 5:** Correlations, Cronbach’s alpha, McDonald’s omega, composite reliability, average variance extracted (AVE), AVE square roots, and mean and standard deviation of SOI-R.

	Pearson’s Correlations								
	0	1	2	3	α	ω	CR	AVE	Mean (*SD*)
0. Total	**0.707**				0.823	0.836	0.869	0.500	2.60 (1.40)
1. Sociosexual behavior	0.615 **	**0.815**			0.742	0.857	0.850	0.664	0.84 (1.15)
2. Sociosexual attitude	0.911 **	0.430 **	**0.848**		0.798	0.801	0.884	0.719	4.42 (2.50)
3. Sociosexual desire	0.756 **	0.226 **	0.520 **	**0.868**	0.830	0.839	0.902	0.754	2.53 (1.63)

** *p* < 0.001; α = Cronbach’s alpha; ω = McDonald’s omega; CR = composite reliability; AVE = average variance extracted; bold (diagonal) = AVE square roots; *SD* = Standard deviation.

**Table 6:** Correlations between FoIS and SOI-R.

	SOI-R Total	Sociosexual Behavior	Sociosexual Attitude	Sociosexual Desire
FoIS total	0.092 *	−0.053	0.049	0.195 **
Imagined Fear of Closeness	0.112 **	−0.082 *	0.110 **	0.177 **
Imagined Openness	−0.018	−0.061	−0.060	0.071
Past Fear of Closeness	0.211 **	0.092 *	0.152 **	0.267 **

Note: ** *p* < 0.001; * *p* < 0.01.

## Data Availability

The data associated with these results are available to those interested, and can be requested from the corresponding author of the study, due to privacy reasons.

## Appendix A

**Fear-of-Intimacy Scale—original version** [20] **(Descutner & Thelen, 1991)**

Part A Instructions: Imagine you are in a close, dating relationship. Respond to the following statements as you would if you were in that close relationship. Rate how characteristic each statement is of you on a scale of 1 to 5 as described below, and put your responses on the answer sheet.

1 Not at all characteristic of me

2 Slightly characteristic of me

3 Moderately characteristic of me

4 Very characteristic of me

5 Extremely characteristic of me

1. I would feel uncomfortable telling 0 about things in the past that I have felt ashamed of.

2. I would feel uneasy talking with 0 about something that has hurt me deeply.

3. I would feel comfortable expressing my true feelings to 0.

4. If 0 were upset I would sometimes be afraid of showing that I care.

5. I might be afraid to confide my innermost feelings to 0.

6. I would feel at ease telling 0 that I care about him/her.

7. I would have a feeling of complete togetherness with 0.

8. I would be comfortable discussing significant problems with 0.

9. A part of me would be afraid to make a long-term commitment to 0.

10. I would feel comfortable telling my experiences, even sad ones, to O.

11. I would probably feel nervous showing 0 strong feelings of affection.

12. 1 would find it difficult being open with 0 about my personal thoughts.

13. I would feel uneasy with 0 depending on me for emotional support.

14. I would not be afraid to share with 0 what I dislike about myself.

15. I would be afraid to take the risk of being hurt in order to establish a closer relationship with 0.

16. I would feel comfortable keeping very personal information to myself.

17. I would not be nervous about being spontaneous with 0.

18. I would feel comfortable telling 0 things that I do not tell other people.

19. I would feel comfortable trusting 0 with my deepest thoughts and feelings.

20. I would sometimes feel uneasy if 0 told me about very personal matters.

21. I would be comfortable revealing to 0 what I feel are my shortcomings and handicaps.

22. I would be comfortable with having a close emotional tie between us.

23. I would be afraid of sharing my private thoughts with 0.

24. I would be afraid that I might not always feel close to 0.

25. I would be comfortable telling 0 what my needs are.

26. I would be afraid that 0 would be more invested in the relationship than I would be.

27. I would feel comfortable about having open and honest communication with 0.

28. I would sometimes feel uncomfortable listening to O’s personal problems.

29. I would feel at ease to completely be myself around 0.

30. I would feel relaxed being together and talking about our personal goals.

Part B Instructions: Respond to the following statements as they apply to your past relationships. Rate how characteristic each statement is of you on a scale of 1 to 5 as described in the instructions for Part A.

31. I have shied away from opportunities to be close to someone.

32. I have held back my feelings in previous relationships.

33. There are people who think that I am afraid to get close to them.

34. There are people who think that I am not an easy person to get to know.

35. I have done things in previous relationships to keep me from developing closeness.

**Escala de Medo da Intimidade—Portuguese version**

Instruções da Parte A: Imagine que está numa relação próxima, de namoro. Responda às afirmações seguintes como responderia se estivesse nessa relação próxima. Avalie o quanto cada afirmação é característica de si numa escala de 1 a 5, conforme descrito abaixo, e coloque as suas respostas na folha de respostas.

1 Nada característico de mim

2 Pouco característico de mim

3 Moderadamente característico de mim

4 Muito característico de mim

5 Extremamente característico de mim

1. Eu sentir-me-ia desconfortável em contar a 0 coisas do meu passado das quais me senti envergonhado(a).

2. Eu sentir-me-ia desconfortável em falar com 0 sobre algo que me magoou profundamente.

3. Eu sentir-me-ia à vontade em expressar os meus verdadeiros sentimentos a 0.

4. Se 0 estivesse aborrecido(a), às vezes eu teria receio de mostrar que me importo.

5. Eu poderia ter receio de confiar a 0 os meus sentimentos mais íntimos.

6. Sentir-me-ia à vontade em dizer a 0 que me importo com ele/ela.

7. Eu teria uma sensação de total união com 0.

8. Sentir-me-ia confortável em discutir problemas significativos com 0.

9. Parte de mim teria receio de estabelecer um compromisso de longo prazo com 0.

10. Sentir-me-ia confortável em contar a 0 as minhas experiências, mesmo as tristes.

11. Provavelmente sentir-me-ia nervoso(a) ao mostrar a 0 sentimentos fortes de afeto.

12. Teria dificuldade em ser aberto(a) com 0 sobre os meus pensamentos pessoais.

13. Sentir-me-ia desconfortável se 0 dependesse de mim emocionalmente.

14. Eu não teria receio de partilhar com 0 aquilo de que não gosto em mim.

15. Teria receio de correr o risco de me magoar para estabelecer uma relação mais próxima com 0.

16. Sentir-me-ia confortável em manter informações muito pessoais para mim.

17. Não ficaria nervoso(a) em ser espontâneo(a) com 0.

18. Sentir-me-ia à vontade para contar a 0 coisas que não conto a outras pessoas.

19. Sentir-me-ia confortável em confiar a 0 os meus pensamentos e sentimentos mais profundos.

20. Por vezes sentir-me-ia desconfortável se 0 me contasse assuntos muito pessoais.

21. Eu sentir-me-ia confortável em revelar a 0 o que considero serem as minhas falhas e limitações.

22. Eu sentir-me-ia confortável em ter um vínculo emocional próximo entre nós.

23. Teria receio de partilhar os meus pensamentos privados com 0.

24. Teria receio de que pudesse nem sempre sentir-me próximo(a) de 0.

25. Sentir-me-ia confortável em dizer a 0 quais são as minhas necessidades.

26. Teria receio de que 0 pudesse estar mais envolvido(a) na relação do que eu.

27. Sentir-me-ia confortável em ter uma comunicação aberta e honesta com 0.

28. Por vezes, sentir-me-ia desconfortável ao ouvir os problemas pessoais de 0.

29. Sentir-me-ia à vontade para ser completamente eu próprio(a) com 0.

30. Sentir-me-ia relaxado(a) em estar junto e falar sobre os nossos objetivos pessoais.

Instruções da Parte B: Responda às afirmações seguintes com base nas suas relações passadas. Avalie o quanto cada afirmação é característica de si numa escala de 1 a 5, conforme descrito nas instruções da Parte A.

31. Afastei-me de oportunidades de estar próximo(a) de alguém.

32. Retive os meus sentimentos em relações anteriores.

33. Há pessoas que acham que tenho medo de me aproximar delas.

34. Há pessoas que acham que não sou fácil de conhecer.

35. Em relações anteriores, fiz coisas para me impedir de desenvolver proximidade.

## References

[1] Silva R.C.P.C.D., Amaral A.C.S., Quintanilha A.K.S., Almeida V.A.R.D., Rodrigues M.V.F., Oliveira A.J., Morgado F.F.D.R. (2021). Cross-cultural adaptation of body image assessment instruments for university students: A systematic review. Psicol. Reflexão Crítica.

[2] Loewenthal K.M., Lewis C.A. (2020). An Introduction to Psychological Tests and Scales.

[3] Streiner D.L., Norman G.R., Cairney J. (2024). Health Measurement Scales: A Practical Guide to Their Development and Use.

[4] Luong R., Flake J.K. (2023). Measurement invariance testing using confirmatory factor analysis and alignment optimization: A tutorial for transparent analysis planning and reporting. Psychol. Methods.

[5] Chen F.F. (2007). Sensitivity of goodness of fit indexes to lack of measurement invariance. Struct. Equ. Model..

[6] Arnett J.J. (2006). Emerging adulthood in Europe: A response to Bynner. J. Youth Stud..

[7] Hardesty M., Wilson S.E., Wasserman L., Young S., Massey S., Merriwether A. (2024). What Are College Students Talking About When They Say They’re “Just Talking?”. Emerg. Adulthood.

[8] Weaver S.J., Herold E.S. (2000). Casual sex and women: Measurement and motivational issues. J. Psychol. Hum. Sex..

[9] Hamilton L., Armstrong E.A. (2009). Gendered sexuality in young adulthood: Double binds and flawed options. Gend. Soc..

[10] Luz R., Alvarez M.J., Godinho C.A., Pereira C.R. (2022). A fertile ground for ambiguities: Casual sexual relationships among Portuguese emerging adults. Front. Psychol..

[11] Kennedy S., Balderrama-Durbin C. (2021). Risky casual sex and posttraumatic stress in college females: An examination of assault history, self-esteem, and social support. Violence Against Women.

[12] Traeen B. (2000). Breaking the speed of loneliness: Sexual partner change and the fear of intimacy. Cult. Health Sex..

[13] Wade L. (2021). Doing casual sex: A sexual fields approach to the emotional force of hookup culture. Soc. Probl..

[14] Erikson E.H. (1993). Childhood and Society.

[15] Bergeron S., Brassard A., Mondor J., Péloquin K. (2020). Under, over, or optimal commitment? Attachment insecurities and commitment issues in relationally distressed couples. J. Sex Marital Ther..

[16] Carreno D.F., Eisenbeck N., Cangas A.J., García-Montes J.M., Del Vas L.G., María A.T. (2020). Spanish adaptation of the Personal Meaning Profile-Brief: Meaning in life, psychological well-being, and distress. Int. J. Clin. Health Psychol..

[17] Prager K.J. (1997). The Psychology of Intimacy.

[18] Moss B.F., Schwebel A.I. (1993). Defining intimacy in romantic relationships. Fam. Relat..

[19] Rokach A. (2024). Love Culturally: How Does Culture Affect Intimacy, Commitment & Love. J. Psychol..

[20] Descutner C.J., Thelen M.H. (1991). Development and validation of a Fear-of-Intimacy Scale. Psychol. Assess..

[21] Origlia G., Limoncin E., Mollaioli D., Sansone A., Colonnello E., Jannini E.A., Ciocca G. (2023). Sociosexuality and Capacity to Love: The Influence of Primary Bonds for Disengaged Sexual Behavior. Sex. Cult..

[22] Yu K., Yu D., Zhou R. (2022). A Short-Term Longitudinal Investigation of Insecure Attachments and Hooking Up Among Chinese College Students. Sex. Res. Soc. Policy.

[23] Khanghah H.B., Samkhaniani E. (2024). Comparison of Cognitive Behavioral Therapy and Mindfulness-Based Therapy on Relational Obsessive-Compulsive Disorder and Fear of Intimacy in Female Students. J. Adolesc. Youth Psychol. Stud..

[24] Reis S., Grenyer B.F.S. (2004). Fear of intimacy in women: Relationship between attachment styles and depressive symptoms. Psychopathology.

[25] Scigala D.K., Fabris M.A., Badenes Ribera L., Zdankiewicz Scigala E., Longobardi C. (2021). Alexithymia and Self-Differentiation: The Role of Fear of Intimacy and Insecure Adult Attachment. Contemp. Fam. Ther..

[26] Sakman E., Urganci B., Sevi B. (2021). Your cheating heart is just afraid of ending up alone: Fear of being single mediates the relationship between attachment anxiety and infidelity. Pers. Individ. Differ..

[27] Rodrigues D., Lopes D. (2017). Sociosexuality, Commitment, and Sexual Desire for an Attractive Person. Arch. Sex. Behav..

[28] Rodrigues D., Lopes D., Pereira M. (2017). Sociosexuality, commitment, sexual infidelity, and perceptions of infidelity: Data from the second love web site. J. Sex Res..

[29] Rodrigues C., Blais M., Lavoie F., Adam B.D., Goyer M.F., Magontier C. (2018). Passion, intimacy, and commitment in casual sexual relationships in a Canadian sample of emerging adults. J. Sex Res..

[30] Simpson J.A., Gangestad S.W. (1991). Individual differences in sociosexuality: Evidence for convergent and discriminant validity. J. Pers. Soc. Psychol..

[31] Urganci B., Sevi B. (2023). Sociosexuality: Infidelity. Encyclopedia of Sexual Psychology and Behavior.

[32] Penke L., Asendorpf J.B. (2008). Beyond global sociosexual orientations: A more differentiated look at sociosexuality and its effects on courtship and romantic relationships. J. Pers. Soc. Psychol..

[33] Alvarez M.J., Pereira C.R., Godinho C.A., Luz R. (2021). Clear-cut terms and culture-sensitive characteristics of distinctive casual sexual relationships in Portuguese emerging adults. Sex. Cult..

[34] Wentland J.J., Reissing E.D. (2011). Taking casual sex not too casually: Exploring definitions of casual sexual relationships. Can. J. Hum. Sex..

[35] Tholander M., Tour N. (2020). Lessons in casual sex: Narratives of young Swedish women. Sex. Cult..

[36] Wongsomboom V., Burleson M.H., Webster G.D. (2020). Women’s orgasm and sexual satisfaction in committed sex and casual sex: Relationship between sociosexuality and sexual outcomes in different sexual contexts. J. Sex. Res..

[37] Wei F., Wang C., Jia Y., Zhang B., Wang W. (2019). How sexual behaviors are influenced by personal cognition and control toward sex? Let Chinese university students tell you. Int. J. Psychoanal..

[38] Weiser D.A., Shrout M.R., Thomas A.V., Edwards A.L., Pickens J.C. (2023). “I’ve been cheated, been mistreated, when will I be loved”: Two decades of infidelity research through an intersectional lens. J. Soc. Pers. Relat..

[39] Garcia J.R., Gesselman A.N., Massey S.G., Seibold-Simpson S.M., Merriwether A.M. (2018). Intimacy through casual sex: Relational context of sexual activity and affectionate behaviors. J. Relat. Res..

[40] Gana K., Arshakyan D. (2023). Relationship between sociosexuality and condom use frequency among young French college students. Eur. J. Psychol..

[41] Laan E.T., Klein V., Werner M.A., van Lunsen R.H., Janssen E. (2021). In pursuit of pleasure: A biopsychosocial perspective on sexual pleasure and gender. Int. J. Sex. Health.

[42] Schmitt D.P. (2005). Sociosexuality from Argentina to Zimbabwe: A 48-nation study of sex, culture, and strategies of human mating. Behav. Brain Sci..

[43] Delgado Amaro H., Alvarez M.J., Ferreira J.A. (2021). Portuguese college students’ perceptions about the social sexual double standard: Developing a comprehensive model for the social SDS. Sex. Cult..

[44] Grello C.M., Welsh D.P., Harper M.S. (2006). No strings attached: The nature of casual sex in college students. J. Sex. Res..

[45] Wesche R., Claxton S.E., Waterman E.A. (2021). Emotional outcomes of casual sexual relationships and experiences: A systematic review. J. Sex. Res..

[46] Ingersoll T.S., Norvilitis J.M., Zhang J., Jia S., Tetewsky S. (2008). Reliability and validity of the fear of intimacy scale in China. J. Pers. Assess..

[47] Segovia A.N., Maxwell J.A., DiLorenzo M.G., MacDonald G. (2019). No strings attached? How attachment orientation relates to the varieties of casual sexual relationships. Personal. Individ. Differ..

[48] Cortoni F., Marshall W.L. (2001). Sex as a coping strategy and its relationship to juvenile sexual history and intimacy in sexual offenders. Sex. Abus. A J. Res. Treat..

[49] Yadav S., Khanam A. (2024). Exploring the effect of casual sexual behavior and emotional intimacy on the self-esteem of young adults. Int. J. Interdiscip. Approaches Psychol..

[50] Greszta E., Jastrzębski J., Izdebski Z., Kowalska-Dąbrowska M., Januszkiewicz A. (2016). Attachment style, love components and sociosexual orientation of men and women in different types of heterosexual relationships. Pol. Forum Psychol..

[51] Wizła M., Lewczuk K. (2024). The Associations Between Attachment Insecurity and Compulsive Sexual Behavior Disorder or Problematic Pornography Use: The Mediating Role of Emotion Regulation Difficulties. Arch. Sex. Behav..

[52] Schachner D.A., Shaver P.R. (2004). Attachment dimensions and sexual motives. Pers. Relatsh..

[53] Townsend J.M., Jonason P.K., Wasserman T.H. (2020). Associations between motives for casual sex, depression, self-esteem, and sexual victimization. Arch. Sex. Behav..

[54] Sherman M., Hackathorn J. (2020). Keeping it casual: Stripping behaviors in non-sex worker populations. Sex. Cult..

[55] Koike M., Loughnan S., Stanton S.C. (2023). Virtually in love: The role of anthropomorphism in virtual romantic relationships. Br. J. Soc. Psychol..

[56] Courtice E.L., Shaughnessy K. (2017). Technology-mediated sexual interaction and relationships: A systematic review of the literature. Sex. Relat. Ther..

[57] Eleuteri S., Terzitta G. (2021). Sexuality during the COVID-19 pandemic: The importance of Internet. Sexologies.

[58] Lehmiller J.J., Garcia J.R., Gesselman A.N., Mark K.P. (2021). Less Sex, but More Sexual Diversity: Changes in Sexual Behavior during the COVID-19 Coronavirus Pandemic. Leisure Sci..

[59] Obeid S., Sacre H., Haddad C., Akel M., Fares K., Zakhour M., Hallit S. (2020). Factors associated with fear of intimacy among a representative sample of the Lebanese population: The role of depression, social phobia, self-esteem, intimate partner violence, attachment, and maladaptive schemas. Perspect. Psychiatr. Care.

[60] Vossler A., Moller N.P. (2020). Internet affairs: Partners’ perceptions and experiences of internet infidelity. J. Sex Marital Ther..

[61] Muniruzzaman M.D. (2017). Transformation of intimacy and its impact in developing countries. Life Sci. Soc. Policy.

[62] Pearce E., Machin A., Dunbar R.I. (2021). Sex differences in intimacy levels in best friendships and romantic partnerships. Adapt. Hum. Behav. Physiol..

[63] Wharton A.S., Erikson R.J. (1993). Managing the emotions on the job and at home: Understanding the consequences of multiple emotional roles. Acad. Manag. Rev..

[64] Spielmann S.S., Nehmeh S., Cantarella I.A. (2023). Worth the risk? Fear of being single and willingness to make risky health decisions in sex and dating contexts. Soc. Personal. Psychol. Compass.

[65] Gaspar M., Grey C., Wells A., Hull M., Tan D.H., Lachowsky N., Grace D. (2022). Public health morality, sex, and COVID-19: Sexual minority men’s HIV pre-exposure prophylaxis (PrEP) decision-making during Ontario’s first COVID-19 lockdown. Crit. Public Health.

[66] Hackathorn J.M., Malm E. (2022). The experience of sex guilt: The roles of parenting, adult attachment, and sociosexuality. Sex. Cult..

[67] Holt-Lunstad J. (2022). Social connection as a public health issue: The evidence and a systemic framework for prioritizing the “social” in social determinants of health. Annu. Rev. Public Health.

[68] Döring N., Krämer N., Mikhailova V., Brand M., Krüger T.H., Vowe G. (2021). Sexual interaction in digital contexts and its implications for sexual health: A conceptual analysis. Front. Psychol..

[69] Hülür G., Macdonald B. (2020). Rethinking social relationships in old age: Digitalization and the social lives of older adults. Am. Psychol..

[70] Doi S.C., Thelen M.H. (1993). The Fear-of-Intimacy Scale: Replication and extension. Psychol. Assess..

[71] Ingersoll T.S., Poulin J., Deng R., Shan X., Witt H., Swain M. (2012). Fear of intimacy with helping professionals scale: Reliability and validity of English and Mandarin versions. J. Evid. Based Soc. Work.

[72] Lyvers M., Edwards M., Thorberg F. (2017). Alexithymia, attachment and fear of intimacy in young adults. J. Psychol. Behav. Sci..

[73] Phillips T.M., Wilmoth J.D., Wall S.K., Peterson D.J., Buckley R., Phillips L.E. (2013). Recollected parental care and fear of intimacy in emerging adults. Fam. J..

[74] Rohner R.P., Filus A., Melendez-Rhodes T., Kuyumcu B., Machado F., Roszak J., Roy K. (2019). Psychological maladjustment mediates the relation between remembrances of parental rejection in childhood and adults’ fear of intimacy: A multicultural study. Cross-Cult. Res..

[75] Senese V.P., Miranda M.C., Lansford J.E., Bacchini D., Nasti C., Rohner R.P. (2020). Psychological maladjustment mediates the relation between recollections of parental rejection in childhood and adults’ fear of intimacy in Italy. J. Soc. Pers. Relat..

[76] Sherman M.D., Thelen M.H. (1996). Fear of Intimacy Scale: Validation and extension with adolescents. J. Soc. Pers. Relat..

[77] Ashcroft R.E. (2008). The declaration of Helsinki. The Oxford Textbook of Clinical Research Ethics.

[78] Beaton D.E., Bombardier C., Guillemin F., Ferraz M.B. (2000). Guidelines for the process of cross-cultural adaptation of self-report measures. Spine.

[79] Parker C., Scott S., Geddes A. (2019). Snowball sampling. SAGE Res. Methods Found..

[80] Neto F. (2016). Psychometric properties of a Portuguese version of the revised sociosexual orientation inventory. J. Relatsh. Res..

[81] Kline R.B. (2023). Principles and Practice of Structural Equation Modeling.

[82] Schreiber J.B., Nora A., Stage F.K., Barlow E.A., King J. (2006). Reporting structural equation modeling and confirmatory factor analysis results: A review. J. Educ. Res..

[83] Cohen J. (1988). Statistical Power Analysis.

[84] Tabachnick B.G., Fidell L.S. (2014). Using Multivariate Statistics.

[85] Hu L.T., Bentler P.M. (1999). Cutoff criteria for fit indexes in covariance structure analysis: Conventional criteria versus new alternatives. Struct. Equ. Model..

[86] Hooper D., Coughlan J., Mullen M.R. (2008). Structural equation modelling: Guidelines for determining model fit. Struct. Equ. Model..

[87] McNeish D., Wolf M.G. (2023). Dynamic fit index cutoffs for confirmatory factor analysis models. Psychol. Methods.

[88] Cohen I., Huang Y., Chen J., Benesty J., Cohen I., Benesty J. (2009). Pearson correlation coefficient. Noise Reduction in Speech Processing.

[89] Fornell C., Larcker D.F. (1981). Evaluating structural equation models with unobservable variables and measurement error. J. Mark. Res..

[90] Calkins F.C., Gervais S.J., Sáez G., Martin M.J., Davidson M.M., Brock R.L. (2023). An integrated conceptual framework linking attachment insecurity to increased risk for both enacting and experiencing objectification. Psychol. Women Q..

[91] Ka W.L., Bottcher S., Walker B.R. (2020). Attitudes toward consensual non-monogamy predicted by sociosexual behavior and avoidant attachment. Curr. Psychol..

[92] Ribeiro F.N., Sousa-Gomes V., Moreira D., Moreira D.S., Oliveira S., Fávero M. (2022). The Relationship Between Romantic Attachment, Intimacy, and Dyadic Adjustment for Female Sexual Function. Sex. Res. Soc. Policy.

[93] Kansky J., Diener E. (2018). What’s love got to do with it? Romantic relationships and well-being. Handbook of Well-Being.

[94] Jorgensen-Wells M.A., James S.L., Holmes E.K. (2021). Attachment development in adolescent romantic relationships: A conceptual model. J. Fam. Theory Rev..

[95] Hmidan A., Weaver A.D. (2019). Sex dreams: Gender, erotophilia, and sociosexuality as predictors of content, valence, and frequency. Can. J. Hum. Sex..

[96] White J., Lorenz H., Perilloux C., Lee A. (2018). Creative casanovas: Mating strategy predicts using—But not preferring—Atypical flirting tactics. Evol. Psychol. Sci..

[97] Hudson N.W., Fraley R.C. (2017). Adult attachment and perceptions of closeness. Pers. Relatsh..

[98] Rong Z., Wen Z., Maoxu L., Ya L., Song F., Hui W., Yunli Y. (2022). Relationship between childhood sexual abuse and attitudes toward premarital sexual permissiveness among middle school students in Luzhou, China. BMC Public Health.

[99] Koch R., Miles S. (2021). Inviting the stranger in: Intimacy, digital technology and new geographies of encounter. Prog. Hum. Geogr..

